# Red and Far-Red LED Lighting Enhances Protoplast-to-Plant Regeneration in Broccoli (*Brassica oleracea* var. *italica*)

**DOI:** 10.3390/plants15060905

**Published:** 2026-03-14

**Authors:** Miriam Romero-Muñoz, José Manuel Gambín-Sánchez, Francisco José Vidal-Sánchez, José E. Cos-Terrer, Margarita Pérez-Jiménez

**Affiliations:** 1 Department of Plant Biotechnology, Genomics and Breeding, Instituto Murciano de Investigación y Desarrollo Agrario y Alimentario (IMIDA), 30150 Murcia, Spain; josem.gambin@carm.es (J.M.G.-S.); fjose.vidal@um.es (F.J.V.-S.); josee.cos@carm.es (J.E.C.-T.); margarita.perez3@carm.es (M.P.-J.); 2 Department of Biochemistry and Molecular Biology—A, University of Murcia, Regional Campus of International Excellence “Campus Mare Nostrum”, 30100 Murcia, Spain

**Keywords:** biotechnological tools, cell culture, crop breeding, organogenesis, LED spectra

## Abstract

Plants have a remarkable ability to regenerate tissues and organs from single cells, a property that underpins in vitro protoplast regeneration. Efficient protoplast-to-plant regeneration remains a major bottleneck for genome engineering in many crop species, including broccoli (*Brassica oleracea* var. *italica*). In this study, we established and optimized a regeneration system for broccoli cv. Claremont by evaluating enzyme composition, light quality, and culture media at successive stages of development. Among the tested enzyme mixtures, 1.5% Cellulase R-10 combined with 0.4% Macerozyme R-10 yielded the highest protoplast viability and recovery. Alginate-embedded protoplasts were cultured under control (dark), blue, and red + far-red LED illumination. Red + far-red treatment significantly enhanced microcolony formation, plating efficiency, and shoot regeneration compared with blue light, whereas blue illumination consistently reduced regenerative performance. The inclusion of activated charcoal in the regeneration medium further increased shoot production. The generalized linear model analyses identified light quality as a significant predictor of both shoot number and regeneration. To our knowledge, this study provides one of the first demonstrations of LED-assisted enhancement of protoplast regeneration in broccoli. The optimized protocol enables whole-plant recovery within approximately 5 months and offers a practical platform for CRISPR-based genome editing and advanced breeding applications in *B. oleracea*.

## 1. Introduction

Plants possess a remarkable capacity to reprogram differentiated somatic cells and regenerate tissues, organs, or even entire plants from a single cell, reflecting a high degree of developmental plasticity and totipotency [1,2]. This regenerative potential underpins in vitro protoplast regeneration, a multistep process that includes cell wall reconstitution, re-entry into the cell cycle, proliferation of pluripotent callus, and subsequent de novo organogenesis leading to complete plantlets [3,4]. As single, cell wall-free plant cells, protoplasts offer a powerful system for dissecting cellular reprogramming and for implementing advanced genome engineering approaches [5,6]. CRISPR/Cas systems can be delivered into protoplasts using DNA-free strategies based on ribonucleoprotein (RNP) complexes, and protoplast-to-plant pipelines have been successfully established in several species, demonstrating the potential of this platform for precise, transgene-free editing [7,8,9,10]. However, shoot regeneration from edited protoplasts remains inefficient, genotype-dependent, and technically demanding in many crops, and this bottleneck continues to limit the broader use of protoplast-based genome editing and somatic hybridization systems [4,6]. Broccoli (*Brassica oleracea* L. var. *italica*), the target species of this study, is a high-value vegetable whose global consumption has increased due to its nutritional quality and high content of health-promoting phytochemicals. Broccoli heads accumulate substantial levels of vitamins, minerals, glucosinolates, phenolic compounds, and other bioactive molecules, and numerous studies have linked broccoli-derived glucoraphanin and its isothiocyanate hydrolysis product, sulforaphane, with antioxidant, anticancer, cardiometabolic, and anti-inflammatory effects [11,12,13]. At the same time, broccoli production faces increasing challenges from climate change, such as heat and drought episodes and shifting pest and disease pressures, which underscores the need for cultivars with enhanced resilience and stable yield under variable environments [14,15]. Conventional breeding strategies, including the use of cytoplasmic male sterility systems and interspecific hybridization within the *Brassica* genus, have been used to introduce desirable traits, but the cross-pollinated floral biology and reproductive barriers make large-scale F_1_ seed production and the exploitation of distant germplasm cumbersome [16,17]. In this context, efficient protocols for protoplast isolation and regeneration in broccoli would greatly facilitate both CRISPR-based precision breeding and somatic hybridization schemes aimed at developing climate-resilient and high-value cultivars.

Protoplast technology in the *Brassica* genus has a long history, and regenerated plants have been obtained from *B. rapa*, *B. napus*, and *B. oleracea* using various tissues as starting materials (reviewed by [16]). Embedding protoplasts in calcium alginate layers has proven particularly effective in the *Brassica* species, improving cell survival, division frequency, and microcallus formation [4,18,19]. Nevertheless, regeneration efficiency remains strongly genotype-dependent and often low, with shoot formation and rooting still representing major hurdles in several *B. oleracea* varieties, most extensively studied in cabbage (*B. oleracea* var. *capitata*) [4,20,21]. This limitation is particularly restrictive for broccoli, where, unlike other B. oleracea crops, reliable regeneration protocols are still scarce. Consequently, robust protoplast-to-plant systems are needed to bridge transient editing in protoplasts and the recovery of stable edited plants, as well as to enable somatic hybridization and cybrid production in breeding programmes [16]. Alongside chemical and nutritional factors, light has emerged as a key environmental regulator of in vitro plant regeneration. Plants sense light quantity and quality through distinct photoreceptors, including phytochromes (red/far-red), cryptochromes, and phototropins (blue/UV-A), and UVR8 (UV-B), which converge on transcriptional networks involving phytochrome-interacting factors (PIFs) and other regulators to modulate hormone signalling, cell division, stress responses, and morphogenesis [22,23,24]. The advent of light-emitting diode (LED) technology has enabled precise spectral control in tissue-culture systems, and numerous studies have shown that LED-based lighting can enhance callus induction, shoot and root organogenesis, and overall micropropagation efficiency compared with conventional fluorescent lamps [25,26,27]. Red and blue LEDs, alone or in combination, have been reported to differentially regulate shoot multiplication, rooting, plantlet quality, and acclimatization in a range of horticultural crops, highlighting light quality as a central parameter in designing efficient in vitro regeneration protocols [28,29,30]. Beyond morphogenesis per se, LED spectra are powerful elicitors of secondary metabolism, which is increasingly recognized as being tightly linked to cell fate, stress tolerance, and regenerative capacity. Red and blue light or their combinations, can significantly alter phenolic and flavonoid accumulation, antioxidant status, and the production of specialized metabolites in in vitro cultures, including callus systems [29,31,32,33,34,35]. In several medicinal and horticultural species, red light has been shown to promote callus growth while modulating antioxidant enzyme activity and enhancing phenolic and flavonoid contents—traits that often correlate with improved differentiation potential and stress resilience [35,36,37]. These observations suggest that spectral quality not only drives photomorphogenic responses but may also prime metabolic and redox states that favour the transition from undifferentiated callus to organogenic tissues.

Despite the rapidly growing body of work on LEDs in micropropagation and callus cultures, the potential of LED spectral quality to enhance protoplast regeneration has received very limited attention. To date, published studies on LED-mediated protoplast regeneration have focused almost exclusively on brown algae: in commercial kelp, *Undaria pinnatifida*, dichromatic red–blue LEDs significantly improved regeneration from gametophyte-derived protoplasts compared with white light [38,39]. To our knowledge, no work has yet reported the use of LED lighting to promote protoplast regeneration in broccoli or other *Brassica* crops, despite the recognized need to improve regeneration efficiency in these species [3,4,16]. This gap highlights a clear opportunity to integrate light quality as a controllable factor in *Brassica* protoplast systems. In this study, we developed and optimized a regeneration protocol for broccoli protoplasts (cv. Claremont) by refining the enzyme solution for protoplast isolation, optimizing culture media for cell division and microcallus proliferation, evaluating the effects of different LED spectra on protoplast regeneration, and testing multiple shoot induction, multiplication, and rooting media. This improved protocol provides new insights into how light quality interacts with cellular pluripotency and establishes a practical framework for genome engineering and somatic hybridization in broccoli.

## 2. Results

### 2.1. Optimization of Protoplast Isolation Protocol

**Figure 1:**
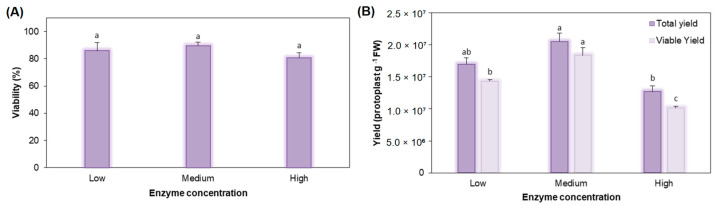
Effect of three enzyme formulations (low, medium and high enzyme solutions) differing in Cellulase R-10 and Macerozyme R-10 concentrations on (**A**) protoplast viability and (**B**) total and viable protoplast yield of broccoli cv. Claremont. All data are expressed as mean ± standard error of the mean (*n* = 4). Different letters among bars indicate significant differences among enzyme solutions according to Duncan’s multiple range test (*p* ≤ 0.05).

To determine the optimal enzyme formulation for protoplast isolation, three enzyme solutions differing in Cellulase R-10 and Macerozyme R-10 concentrations were evaluated for overnight digestion of broccoli cv. Claremont leaves. The low enzyme solution (low ES) contained 1.0% (*w*/*v*) Cellulase R-10 and 0.2% (*w*/*v*) macerozyme R-10; the medium enzyme solution (medium ES) contained 1.5% cellulase and 0.4% macerozyme; and the high enzyme solution (high ES) contained 2.0% cellulase and 1.0% macerozyme. Protoplast viability did not differ significantly among the three enzyme solutions (Figure 1A), indicating that increasing enzyme concentrations within this range did not compromise membrane integrity. In contrast, protoplast yield was significantly affected by the enzyme formulation (Figure 1B). Both total and viable yields were highest under medium ES and declined under high ES. For total yield, medium ES produced 20.6% more protoplasts than low ES and 61.4% more than high ES. Although no significant differences were detected between medium and low ES, medium ES yielded 1.61-fold higher total protoplast numbers than high ES. A similar trend was observed for viable yield, where medium ES produced 28.7% more viable protoplasts than low ES and 82.2% more than high ES (1.82-fold increase relative to high ES). In contrast, viability percentage varied only slightly among treatments (4–11% differences) and showed no statistically significant variation. These results indicate that the medium enzyme solution (1.5% Cellulase R-10 + 0.4% Macerozyme R-10) provides the most favourable balance between efficient cell wall digestion and preservation of protoplast integrity and was therefore selected for all subsequent isolations.

### 2.2. Protoplast Cultivation Under LED Lights

**Figure 2:**
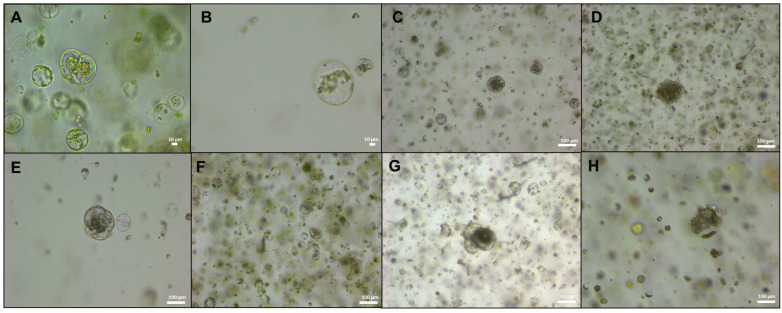
Protoplast division at 3 DAC on medium used for Protocol 1 (**A**) and medium used for Protocol 2 (**B**); microcalli (≤100 μm) formation at 10 DAC under Protocol 1 under dark (**C**), red + far-red light (**D**), and blue light (**E**) exposition; and microcalli formation at 10 DAC under Protocol 2 under dark (**F**), red + far-red light (**G**) and blue light (**H**) exposition. Scale bars as indicated.

**Figure 3:**
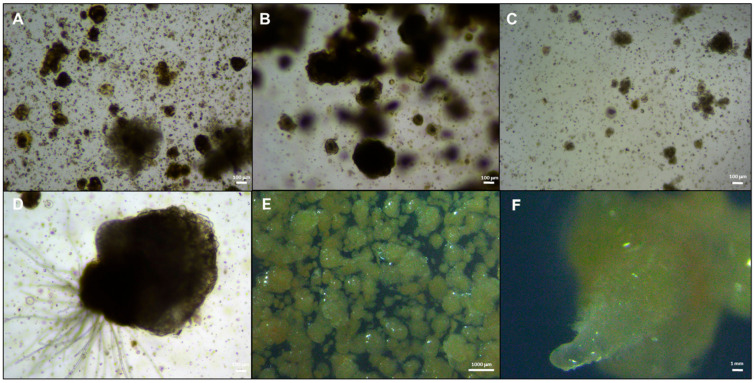
Progression of microcallus formation and early organogenic responses from broccoli protoplast-derived cultures under red + far-red light conditions. Early-stage microcalli (≤100 μm) at 30 DAC obtained under dark (**A**), red + far-red (**B**), and blue light (**C**) exposure; Enlarged microcallus at 45 DAC exhibiting dense cellular organization and early roots (**D**); proliferation phase at 50 DAC showing numerous spherical and compact microcalli with homogeneous size distribution under red + far-red light treatment (**E**); and advanced structure displaying organogenic characteristics, including a protruding meristematic-like dome and differentiated tissue zones (**F**). Scale bars as indicated.

To improve broccoli protoplast regeneration, different culture media and LED light treatments were tested on protoplasts isolated with the optimized enzyme solution. The first cell divisions were observed 3 days after culture (DAC) in all media and light treatments (Figure 2A,B). Small multicellular structures (<8 cells) appeared at 5 DAC in Protocol 1 and at 8 DAC in Protocol 2 under all light regimes (Figure 2). These early clones were visible as compact aggregates derived from single protoplasts and marked the onset of microcallus formation. Microcallus colonies became clearly visible during the second week of culture when protoplasts were cultivated according to Protocol 1 under red + far-red LED treatment or in darkness (control), whereas cultures exposed to blue LED regenerated more slowly and colonies were only evident by the fourth week (Figure 2G and Figure 3C). Under Protocol 2, microcalli appeared at the third week in dark-cultured protoplasts and at the fourth and fifth weeks under red and blue light, respectively. Representative images illustrate the transition from isolated dividing protoplasts to small spherical microcalli and larger, densely structured callus masses, some of which exhibited early organogenic features such as meristematic-like domes and root-like protrusions (Figure 2 and Figure 3).

**Figure 4:**
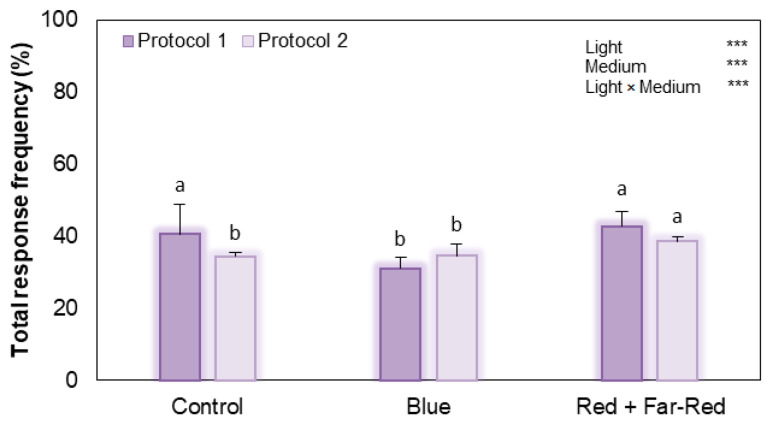
Effect of LED light treatment and culture media (Protocol 1 or 2) on total response frequency at 15 DAC in broccoli cv. Claremont protoplasts. All data are expressed as mean ± standard error of mean (*n* = 5). Different letters among bars indicate significant differences among LED treatments according to Duncan test (*p* ≤ 0.05). Significance levels are coded as ns (non-significant), *** (*p* < 0.001).

Total response frequency assessed at 15 DAC showed that, under Protocol 1, red + far-red light resulted in significantly higher response frequencies than blue light, representing an increase of 1.37-fold. Control conditions were similarly higher (1.31-fold) than blue light, whereas no significant differences were observed between control and red + far-red treatments (Figure 4). Under Protocol 2, red + far-red light resulted in significantly higher response frequency compared with both control and blue treatments, which did not differ from each other. Two-way ANOVA revealed significant effects of culture medium, light quality, and their interaction on total response frequency at 15 DAC (*p* < 0.001 for all factors, Figure 4), indicating that the effect of light depended on the proliferation protocol used. At 45 DAC, colony-formation efficiency (Table 1) under Protocol 1 followed a similar general trend, with the red + far-red and control treatments showing values approximately twofold higher than those of the blue light treatment. Furthermore, the two-way ANOVA revealed no significant main effect of light on colony-formation efficiency across media (Table 1). Similarly, the plating efficiency was markedly reduced under blue light compared to the control and red + far-red conditions in Protocol 1, representing a decrease of approximately 2.0-fold relative to both the control and the red + far-red light conditions. Under Protocol 2, a similar trend was observed with red + far-red light producing significantly higher colony-formation efficiency and plating efficiency (a 2.87-fold increase), whereas control values were statistically intermediate between blue and red + far-red treatments. Importantly, the two-way ANOVA revealed a significant main effect of light quality on plating efficiency (*p* = 0.03), whereas neither the culture medium factor nor the light × medium interaction was significant (Table 1).

### 2.3. De Novo Shoot Regeneration Under LED Treatments

**Figure 5:**
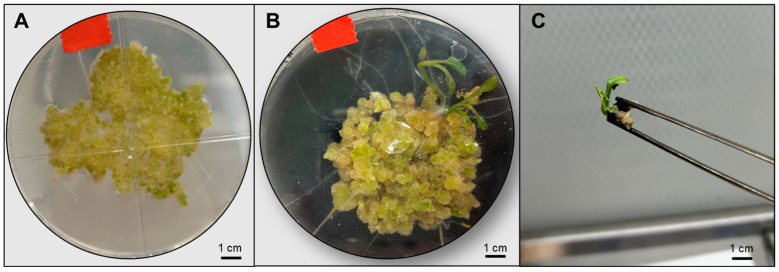
Regeneration response at 100 DAC of broccoli callus cultures under the two tested regeneration media without activated charcoal (**A**) or supplemented with activated charcoal (**B**) in red + far-red treatment; isolated regenerating shoot excised from callus grown in the charcoal-supplemented medium displaying clear shoot differentiation (**C**). Scale bars = 1 cm.

Callus maintained on regeneration medium without activated charcoal showed limited greening and reduced organogenic initiation (Figure 5A), whereas callus cultured on medium supplemented with activated charcoal exhibited enhanced greening, visible shoot primordia, and early shoot development (Figure 5B,C). Shoot regeneration was detected 37 days after transfer to shoot regeneration medium under all light treatments. However, both regeneration percentage and shoot number varied markedly depending on light quality (Table 2). Red + far-red light resulted in the highest regeneration percentage and shoot production, whereas blue light showed the lowest regeneration percentage and shoot number. Regeneration under red + far-red light was approximately 8-fold higher than under blue light and more than 2-fold higher than under control (white) light. Similarly, shoot number under red + far-red light was about 5-fold higher than under blue light and nearly 4-fold higher than under control light. Control light produced intermediate responses. Regarding the culture medium, shoot number was significantly higher in the activated charcoal-supplemented medium compared with the control medium (0.66 vs. 0.15 shoots per explant). Regeneration percentage also tended to be higher in the charcoal-containing medium (45% vs. 14%), although this difference was not statistically significant.

A generalized linear model was used to evaluate the effects of the culture medium and light quality on shoot formation and regeneration. For the shoot number (Poisson distribution, log link), the overall model was significant (Likelihood ratio χ^2^ = 15.289, df = 3, *p* = 0.002). Both culture medium (LR χ^2^ = 6.738, df = 1, *p* = 0.009) and light quality (LR χ^2^ = 8.551, df = 2, *p* = 0.014) significantly affected shoot production. Regeneration (presence/absence of at least one shoot per alginate layer) was analyzed using a generalized linear model with binomial error distribution and logit link function. The model was also significant (LR χ^2^ = 9.091, df = 3, *p* = 0.028). Light quality significantly influenced regeneration probability (LR χ^2^ = 6.640, df = 2, *p* = 0.036), whereas culture medium showed only a marginal effect (*p* = 0.079). Regeneration was the lowest under blue light and the highest under red + far-red light.

### 2.4. In Vitro Multiplication, Rooting, and Acclimatization

**Figure 6:**
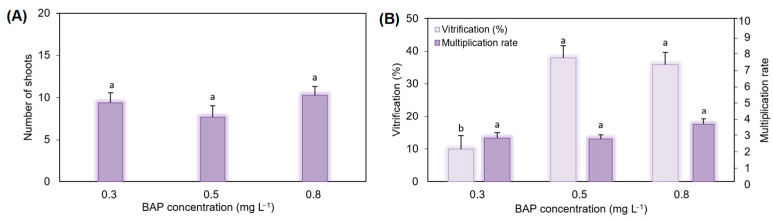
Effect of different concentrations of BAP combined with 0.1 mg L^−1^ IBA on the number of new regenerated shoots (**A**); percentage of vitrification and the multiplication rate (**B**). All data are expressed as mean ± standard error of mean (*n* = 5). Different letters among bars indicate no significant differences between treatments according to Duncan test (*p* ≤ 0.05).

To evaluate the influence of benzylaminopurine (BAP) on shoot multiplication, regenerated explants were cultured on a multiplication medium containing 0.1 mg L^−1^ IBA and different BAP concentrations (0.3, 0.5 and 0.8 mg L^−1^). The number of newly formed shoots, the multiplication rate, and the percentage of vitrification were recorded at the end of the culture period (Figure 6A,B). All BAP concentrations tested supported shoot proliferation, with mean values of newly formed shoots ranging from 7.6 to 10.2. Although slight numerical differences were observed among treatments, statistical analysis indicated that the BAP concentration had no significant effect on the number of new shoots or on the multiplication rate (*p* ≤ 0.05), as all treatments belonged to the same statistical group. In contrast, vitrification was significantly influenced by the BAP concentration. The lowest BAP level (0.3 mg L^−1^) resulted in a significantly lower percentage of vitrified shoots compared with 0.5 and 0.8 mg L^−1^, which did not differ from each other (*p* ≤ 0.05). Specifically, vitrification at 0.3 mg L^−1^ was reduced by approximately 74% and 72% relative to 0.5 and 0.8 mg L^−1^, respectively, indicating that lowering cytokinin concentration markedly decreased hyperhydricity without compromising shoot multiplication.

**Figure 7:**
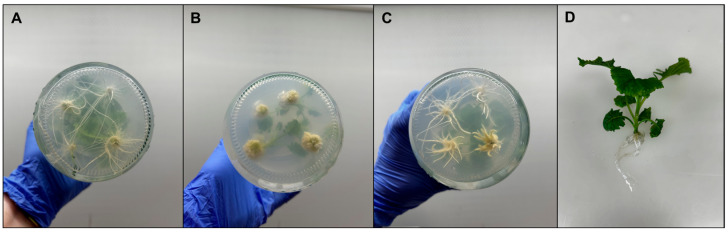
Rooting response of broccoli cv. Claremont regenerated plants under the three tested rooting media: (**A**) without plant hormones (**B**), with 1 mg L^−1^ NAA, (**C**) with 1 mg L^−1^ IBA and regenerated rooting plant (**D**).

For in vitro rooting, regenerated shoots were transferred to three media differing in auxin composition: hormone-free medium, medium supplemented with 1 mg L^−1^ NAA, and medium with 1 mg L^−1^ IBA (Figure 7; Table 3). The explants were kept during 4 weeks in RM. At 10 days after culture, roots started to appear in all media evaluated. Rooting percentage was high in all treatments (100% in hormone-free and NAA-containing media, and 66.67% in IBA), with no significant differences among them (Table 3). By contrast, the number and morphology of roots differed markedly depending on the medium (Figure 7). Shoots cultured on a hormone-free medium developed significantly more roots per explant and the longest roots forming a dense, well-branched root system (Figure 7A,D). In the presence of NAA, rooting percentage remained 100%, but plantlets produced fewer roots that were short and thin and often associated with callus-like, highly “hairy” root structures (Figure 7B). IBA-treated shoots showed the poorest rooting response overall, with the lowest number of roots and short primary roots that were thick and poorly branched, lacking secondary roots (Figure 7C). Rooted plantlets from the hormone-free medium displayed the most vigorous and morphologically normal root systems, which facilitated handling and acclimatization and were therefore successfully transferred to ex vitro conditions.

## 3. Discussion

The results of this study highlight light quality as a critical and previously underexploited factor in protoplast regeneration, particularly the use of red LED illumination, and they represent an important improvement in tissue culture methods for *Brassica oleracea* var. *italica*. We established an efficient protoplast-to-plant regeneration system for broccoli and showed that red LED light acts as a strong promoter of early cell division, microcolony proliferation, and shoot differentiation. While enzyme composition has traditionally been regarded as the primary determinant of successful protoplast isolation, our results demonstrate that physical culture conditions, especially light quality, play an equally crucial role in determining regeneration efficiency. The optimized enzyme mixture (1.5% Cellulase R-10 + 0.4% Macerozyme R-10) yielded high numbers of viable protoplasts, which is in agreement with recent work in cabbage, indicating that intermediate cellulase levels achieve the best balance between wall digestion and membrane integration [19,40]. However, the main innovation of the present work lies in the demonstration that red LED light markedly enhances protoplast regeneration, making this, to our knowledge, the first experimental evidence in *Brassica* linking red LED illumination with enhanced protoplast-derived plant regeneration in *Brassica* crops, in contrast to previous protocols that did not manipulate light spectra as a key variable [4,18,20,40]. This conclusion is supported by our GLM analyses, which identified light quality as a significant predictor of both shoot number (Poisson GLM) and regeneration percentage (binomial GLM), whereas the medium effect was significant for shoot number and only marginal for regeneration, and no significant light × medium interaction was detected. The promotive effect of red + far-red light is likely linked to its capacity to modulate secondary metabolism, redox homeostasis, and photomorphogenic signalling, factors that are increasingly recognized as closely associated with cell competence for differentiation. Red and far-red wavelengths are perceived primarily through the phytochrome system, which dynamically interconverts between the inactive Pr and active Pfr forms depending on the R:FR ratio. The modulation of this balance is known to influence cell cycle regulation, hormonal responsiveness, and meristem activity, all of which are critical determinants of organogenic competence in vitro [23,41]. Light regulates diverse biosynthetic pathways for phenolics, flavonoids, terpenoids, and glucosinolates via photoreceptor-driven signalling networks [42,43]. In vitro studies have consistently shown that specific LED spectra can enhance biomass production and the accumulation of bioactive compounds. For example, monochromatic red or red–blue LEDs significantly improved biomass and secondary metabolite content in callus cultures of *Withania somnifera* and *Eclipta alba*, often accompanied by altered antioxidant enzyme activities and reactive oxygen species (ROS) status [36,37,44]. More broadly, phytochrome-mediated red/far-red signalling is a central regulator of photomorphogenesis and developmental transitions that influence cell proliferation and organogenic competence [23,45]. Notably, under comparable photon flux densities to those used in the present study, enhanced adventitious shoot regeneration under red + far-red LED combinations has been reported in callus cultures of peach cultivars and hybrid rootstocks [46]. Although that study examined organogenesis from callus rather than protoplast-derived tissues, the similar response under comparable irradiance conditions suggests that spectral composition may influence key developmental transitions common to different in vitro regeneration pathways.

In our system, the earlier appearance of microcolonies under red + far-red light, their higher plating and colony-formation efficiencies, together with their higher shoot regeneration suggest that red and far-red wavelengths create a physiological environment favourable for cell-cycle re-entry and organogenic competence, potentially by improving metabolic status and controlling oxidative stress. From a cellular perspective, protoplast regeneration requires extensive transcriptional reprogramming and re-establishment of meristematic competence. Light-regulated signalling pathways may facilitate this transition by coordinating redox homeostasis, auxin–cytokinin balance, and the activation of the developmental regulators associated with shoot meristem initiation [47]. The significant effects of light quality, culture medium, and their interaction on total response frequency detected by two-way ANOVA further indicate that early protoplast competence is shaped by both spectral and nutritional cues. This interaction suggests that the promotive effect of red + far-red light during the initial stages of culture depends, at least partially, on the metabolic context provided by the proliferation medium. Such responses may reflect the well-known crosstalk between light signalling and hormone-regulated developmental pathways [45]. Phytochrome-mediated signalling can influence auxin and cytokinin networks that regulate cell division and organogenic competence. Consequently, differences in hormonal composition and metabolic context between proliferation media may modify how protoplast-derived cells respond to specific light spectra during early regeneration stages [25,26]. By contrast, blue light is frequently associated with stress responses, elevated ROS, and reduced proliferation in tissue culture, consistent with the markedly diminished microcolony formation and regeneration that we observed under blue LEDs [43,48]. In line with this, blue light perception through cryptochromes/phototropins has been linked to ROS-related signalling and stress-associated responses in plants [49], which may further constrain already stress-prone protoplast cultures. From a developmental perspective, red light is known to act via phytochrome-mediated signalling pathways that influence cell expansion, hormonal crosstalk, and meristem activation [22,23]. Recent reviews have emphasized that light is not simply a growth factor but a central regulator of in vitro plant regeneration, affecting auxin-driven callus formation, shoot meristem induction, and phase transitions through coordinated changes in hormone signalling, ROS networks, and gene expression (reviewed by [50]). Our findings are consistent with this view and extend it to protoplast-derived tissues: red + far-red LED light not only maintained early cell division but also enhanced the subsequent transition to shoot formation, particularly when combined with activated charcoal. However, because the light × medium interaction was not significant in our GLMs, the promotive effect of red + far-red illumination appears to act largely independently of the culture medium, while activated charcoal primarily increased shoot output rather than regeneration probability. It should be noted that the photon flux density (PPFD) differed among LED treatments, with red + far-red light providing a higher PPFD than white or blue light. Although this difference in irradiance may have contributed to the enhanced response observed under red + far-red conditions, the magnitude and direction of the biological effects, particularly the clear distinction between red + far-red and white light treatments, suggest that spectral quality rather than photon flux alone played a predominant role in modulating regeneration. Similar observations have been reported in several in vitro culture systems, where spectral composition significantly influenced morphogenesis and organogenic responses through photoreceptor-mediated signalling and hormonal regulation [25,26]. These effects are commonly attributed to the activation of specific photoreceptor-mediated signalling pathways that regulate hormonal balance, metabolic activity, and developmental competence during plant regeneration. Nevertheless, future experiments under equalized PPFD conditions would be valuable to fully disentangle spectral and intensity effects. Activated charcoal likely contributes by adsorbing inhibitory phenolics and excess hormones [51,52], while red light promotes a metabolically favourable and less stressful environment, as reported for several in vitro systems where red LEDs increased biomass and phenolic or antioxidant accumulation and reduced oxidative stress markers [37,53,54], together supporting sustained proliferation and organogenic differentiation.

Regarding cytokinin effects, the lack of significant differences in shoot multiplication across the tested BAP concentrations suggests that, once protoplast-derived shoots are established, the exact cytokinin level within this range becomes less critical than the earlier culture conditions, including the light regime. This is consistent with studies in the *Brassica genus* and other horticultural species, showing that light quality and the conditions used during the initial regeneration phases exert a major influence on shoot proliferation and morphogenesis, often having a stronger impact than moderate changes in cytokinin concentration within a given range [26,55,56]. However, in our work, we observed a lower incidence of hyperhydric (vitrified) tissues when regenerated shoots were cultured at the lowest BAP concentration (0.3 mg L^−1^), indicating that reducing cytokinin levels can mitigate vitrification. This is in line with previous reports showing that high cytokinin concentrations, particularly BAP, are associated with increased hyperhydricity, and that lowering cytokinin levels is an effective strategy to reduce this disorder [57,58]. Similarly, the higher rooting efficiency in the hormone-free medium supports the notion that excessive exogenous auxin can inhibit rooting in *Brassica* and that protoplast-derived shoots may retain sufficient endogenous auxin to initiate roots without supplementation. This aligns with reports that simpler hormonal environments often promote root induction and plantlet quality in the *Brassica* tissue culture [4,20]. Overall, this study underscores the value of integrating light quality as a controllable, biologically meaningful parameter in protoplast culture systems. In our protocol, red + far-red LED light functioned not merely as a neutral or maintenance condition but as an active promoter of differentiation, enhancing both early proliferation and shoot regeneration. Given the long-standing bottleneck of low and genotype-dependent regeneration efficiency in *Brassica* protoplast systems [4,19,40], our findings represent an important shift in protocol design. Because protoplast-based regeneration is a key platform for genome editing and somatic hybridization in *Brassica*, improvements in regeneration frequency directly translate into increased transformation and editing efficiency [47]. Enhancing early cell proliferation and shoot formation through spectral optimization may therefore have immediate implications for CRISPR/Cas-mediated gene editing systems in *B. oleracea*. Future work should build on these results by quantifying secondary metabolite profiles, antioxidant capacity, and ROS status in protoplast-derived microcolonies under different LED spectra, as well as by analyzing the expression of genes involved in cell cycle control, phytochrome signalling, and hormone biosynthesis or response. In particular, linking red/far-red responsiveness to phytochrome-regulated transcriptional programmes and ROS homeostasis during protoplast culture would help clarify the mechanistic basis for the observed improvements in regeneration. It will also be essential to test this protocol across multiple *Brassica* genotypes to determine whether sensitivity to red LED illumination is cultivar-specific or broadly conserved. By establishing light quality and particularly red LED illumination as a key driver of differentiation, the present protocol not only improves practical regeneration outcomes but also opens mechanistic avenues with direct implications for editing efficiency, somatic hybridization, and other biotechnological applications in *B. oleracea* var. *italica*.

## 4. Materials and Methods

### 4.1. Plant Material and Growth Conditions

The study was conducted on the commercial broccoli cv. Claremont (Bejo Ibérica, Spain). Broccoli seeds were sterilized in a solution of 2% (*v*/*v*) sodium hypochlorite and 0.1% (*v*/*v*) Tween 20 for 90 min and washed three times in a laminar flow hood with sterile distilled water for 5 min. The seeds were germinated on the Murashige and Skoog (MS) medium with vitamins [59] (Duchefa Biochemie, Haarlem, The Netherlands) supplemented with 3% (*w*/*v*) sucrose (Duchefa) and 0.7% (*w*/*v*) plant agar (Duchefa). The pH was adjusted to 5.8 using 1 M KOH or 1 M HCl and sterilized through autoclaving at 121 °C for 21 min. In order to obtain plantlets with well-developed leaves, the seeds were placed in sterile culture tubes containing 12 mL of the previously mentioned medium and kept at 25  ±  1 °C under a 16 h light (45 μmol m^−2^ s^−1^; Sylvania, Surrey, UK) photoperiod during 5 weeks.

### 4.2. Experiment 1: Protoplast Isolation Procedure

Newly expanded young leaves from 5-week-old plants were used for protoplast isolation. A total of 0.8–1 g of plant tissue was placed in a Petri dish containing 0.5 M mannitol at pH 5.8 (preplasmolysis solution), cut into small pieces, and incubated for 1 h in the dark at room temperature (RT). Three types of enzyme solutions (ES) were used, differing in enzyme concentration: low ES, which contained 1.0% (*w*/*v*) Cellulase R-10 and 0.2% (*w*/*v*) Macerozyme R-10; medium ES, which contained 1.5% (*w*/*v*) Cellulase R-10 and 0.4% (*w*/*v*) Macerozyme R-10; and high ES, which contained 2.0% (*w*/*v*) Cellulase R-10 and 1.0% (*w*/*v*) Macerozyme R-10. To prepare the enzyme solutions, the powdered enzymes were dissolved in a MMC salt solution (10 mM 2-morpholinoethanesulfonic acid monohydrate (MES), 0.47 M mannitol, 10 mM calcium chloride) according to [60]. All enzyme solutions were filter-sterilized using a 0.22 μm syringe filter (Pall Corporation, New York, USA). For protoplast digestion, preplasmolysed explants were incubated in dark conditions in both enzyme solutions for 16 h with gentle shaking (45 rpm) at room temperature (RT). Briefly, after the incubation period, the isolated protoplasts were filtered through 100 μm cell strainers (pluriSelect Life Science, Leipzig, Germany) to remove undigested tissue and debris. The protoplasts were then collected by centrifugation at 120× *g* for 7 min at RT. The protoplast pellet was resuspended in 2 mL of MMC solution, and the suspension was overlaid on 6 mL of 0.6 M sucrose and centrifuged at 80× *g* for 10 min. Protoplasts, now located at the interphase between the two solutions, were gently collected into a new tube and further purified by adding the MMC solution. This was followed by centrifugation at 120× *g* for 5 min at RT. The purified protoplasts were washed twice with 0.5 M mannitol through centrifugation at 120× *g* for 5 min at RT. The final preparation was routinely examined microscopically to verify the absence of intact cells or tissue debris; only spherical, cell wall-free protoplasts were observed. After resuspension in 0.5 M mannitol, the protoplast yield was counted under a light microscope using a hemocytometer. The protoplast density was adjusted to 8 × 10^5^ protoplasts mL^−1^. Protoplast viability was assessed on the day of purification by staining with fluorescein diacetate (FDA), according to [61]. Metabolically active protoplasts were visualized by excitation of the accumulated fluorescein under blue light at 485 nm, using a 515 nm barrier filter. Observations were made using a Leica DM IL LED inverted microscope (Leica Microsystems GmbH, Wetzlar, Germany). Each enzymatic treatment was performed using four independent biological replicates. Protoplast yield was expressed as the number of protoplasts per gram of fresh weight (FW) of initial tissue. Viability was scored as the percentage of protoplasts exhibiting green fluorescence, with viable protoplasts showing yellow-green fluorescence, while non-viable protoplasts remained invisible.

### 4.3. Experiment 2: Protoplast Embedding and Culture Under LED Treatments

Immobilization of broccoli protoplasts in Ca^2+^-alginate layers was performed following the method found in [62], with several modifications. Protoplast suspension was mixed with an equal volume of sodium alginate solution to obtain a final density of 4 × 10^5^ protoplasts mL^−1^. Then, 2 mL protoplast–alginate mixture was gently spread onto CaCl_2_ -agar in a 55 mm Petri dish. After 1 h incubation at RT, a layer of alginate hydrogel containing immobilized protoplasts was formed. Then, 2 mL CaCl^2^ 50 mM solution was applied onto the alginate hydrogel and incubated for 30 min to complete polymerization. We tested two different protoplast culture protocols differing in hormonal balance (auxin- vs. cytokinin-enriched) to evaluate which formulation better supported early protoplast division and colony development in broccoli. Following Protocol 1, alginate layers were cultured in 55 mm Petri dishes with 10 mL of protoplast proliferation medium 1 (PPM-1) which consisted of Gamborg B5 medium with vitamins [63], 3% (*w*/*v*) sucrose, 6% (*w*/*v*) myo-inositol, 2 mg L^−1^ 6-Benzylaminopurine (BAP), and 0.5 mg L^−1^ naphthaleneacetic acid (NAA), following the formulation described in [60]. Regarding Protocol 2, protoplast proliferation medium 2 (PPM-2) consisted of a half of Gamborg B5 medium with vitamins [63], 2% (*w*/*v*) glucose, 7% (*w*/*v*) D-mannitol, 1 mg L^−1^ BAP, 1 mg L^−1^ NAA, and 0.25 mg L^−1^ 2,4-Dichlorophenoxyacetic acid (2,4-D). The pH was adjusted to 5.8 using 1 M KOH or 1 M HCl, and sterilized by autoclaving at 121 °C for 21 min. Both media were refreshed 7 days after protoplast culture (DAC). After 14 DAC, the alginate layers were transferred to callus proliferation medium (CPM), which was identical to the PPM formulation but without myo-inositol in the case of Protocol 1 and in the case of Protocol 2 the amount of D-mannitol was reduced at 40 g L^−1^. The pH was adjusted to 5.8 and both media were sterilized as previously described. Liquid media were renewed every 10 days. All media are listed in Supplementary Table S1. Each light treatment consisted of five Petri dishes (biological replicates), with one alginate layer per dish. For both media tested, control group was cultured in complete darkness at 25 ± 1 °C; a second group was cultured in 55% red (600–700 nm) + 45% far red light (700–800 nm) (red + far-red), and the third group was cultured in 100% blue light (blue; 400–500 nm). The photosynthetically active photon flux density (PPFD, 400–700 nm) measured at the culture level was 52.58 μmol m^−2^ s^−1^ under blue light (B), 93.64 μmol m^−2^ s^−1^ under red + far-red light (R + FR). The spectral composition and photon flux density parameters were quantified for each of the LED treatments. LED lighting was provided in a controlled climate chamber (Aralab 1200 PLH LED, Madrid, Spain) with a 16 h photoperiod at 25 ± 1 °C.

### 4.4. Experiment 3: Application Under LED Light on Shoot Regeneration

After 60 days of culture (DAC), alginate layers containing microcalli were transferred to solid media for de novo shoot regeneration. Two types of shoot regeneration media (SRM) were used, differing only in the presence or absence of 2.5 g L^−1^ activated charcoal (Panreac applichem, Darmstadt, Germany). The SRM was based on two-thirds strength Murashige and Skoog (MS) medium with vitamins [59] (Duchefa Biochemie, Haarlem, The Netherlands), supplemented with 3% (*w*/*v*) sucrose, 2 mg L^−1^ 6-benzylaminopurine (BAP), 0.1 mg L^−1^ indole-3-acetic acid (IAA), and 0.7% (*w*/*v*) plant agar (all from Duchefa Biochemie). The pH was adjusted to 5.8 using 1 M KOH or 1 M HCl, and the medium was sterilized through autoclaving at 121 °C for 21 min. When indicated, 2.5 g L^−1^ of activated charcoal was added to the medium prior to sterilization. Alginate layers were incubated at 25 °C under white LED light (control; 380–780 nm) or under the same LED treatments previously described: blue LED light (400–500 nm) and red LED light composed by 55% red light (600–700 nm) and 45% far red light (700–800 nm) to promote callus differentiation and shoot formation. Plates were randomly assigned to the three independent light treatments. The PPFD (400–700 nm) measured at the culture level was 74.68 μmol m^−2^ s^−1^ under white light (control), 52.58 μmol m^−2^ s^−1^ under blue light (B), and 93.64 μmol m^−2^ s^−1^ under red + far-red light (R + FR). LED lighting was provided in a controlled climate chamber (Aralab 1200 PLH LED, Madrid, Spain) with a 16 h photoperiod at 25 ± 1 °C. The total number of shoots was counted for each regenerating layer at the end of the culture period. Thus, shoot regeneration rate was calculated using each alginate layer as the experimental unit. Regeneration was considered positive when at least one visible shoot developed on the layer by the end of the culture period. Regeneration efficiency was assessed by determining the percentage of layers that produced shoots. The layers that developed only roots without shoot formation were not considered regenerated.

### 4.5. Experiment 4: Optimization of Multiplication and Rooting Media

After the regenerated shoots appeared, they were excised, and transferred to three different multiplication media consisting of DKW/Juglans medium including vitamins supplemented with 2% (*w*/*v*) sucrose, 0.7% (*w*/*v*) plant agar, 0.1 mg L^−1^ Indole-3-butyric acid (IBA) and different concentration of BAP: 0.8, 0.5, or 0.3 mg L^−1^ BAP. The pH was adjusted to 5.8 using 1 M KOH or 1 M HCl, and the medium was sterilized by autoclaving at 121 °C for 21 min. Regenerated plants were cultured under white LED light under the same environmental conditions described in Section 4.1 (16 h photoperiod, 45 μmol m^−2^ s^−1^, 25 ± 1 °C). After 4 weeks, the multiplication, rate number of shoots, and percentage of vitrification were recorded. Each multiplication treatment consisted of five glass jars (biological replicates), each containing four explants (*n* = 5 biological replicates per treatment). The multiplication rate was calculated as the number of newly formed shoots per initial explant cultured. Vitrification was expressed as the percentage of vitrified explants relative to the total number of explants per treatment. After the plant multiplication phase, three different rooting media (RM) were evaluated. The RM was based on half-strength Murashige and Skoog (MS) medium with vitamins [59] (Duchefa Biochemie, Haarlem, The Netherlands), supplemented with 1% (*w*/*v*) sucrose and 0.7% (*w*/*v*) plant agar. The RM differed in their hormonal concentrations, RM-1 had no growth regulators; RM-2 contained 1 mg L^−1^ IBA, and RM-3 contained 1 mg L^−1^ NAA. For rooting evaluation, five glass jars per treatment were used, each containing four shoots (*n* = 5 biological replicates per treatment). The percentage of rooting, root length, and number of roots per shoot were recorded 4 weeks after culture. The rooted plantlets were transferred to soil and grown further at 23 °C under long day (LD) photoperiod and 100 μmol photons m^−2^ s^−1^ light intensity.

### 4.6. Data Collection and Statistical Analysis

The cultures were observed weekly using a Leica DM IL LED inverted microscope (Leica Microsystems, Wetzlar, Germany). The images were captured with a Leica DFC450C camera and analyzed using the Leica Enersight v1.0.2.97 software (Leica Microsystems). At 15 days after culture (DAC), total response frequency (%) was calculated following [64], as the percentage of responsive protoplast-derived units relative to the total number of viable protoplasts cultured. Plating efficiency (%) was calculated following [65], as the percentage of microcolonies relative to the total number of observed cells (divided and undivided) within the evaluated area. Colony-formation efficiency (%) was calculated as the percentage of total colonies (microcolonies <100 μm + colonies >100 μm) relative to the total number of observed cells within the same microscopic fields. Microscopic observations were carried out on 100–200 cells per layer. During the shoot regeneration phase, regeneration (%) was defined as the percentage of alginate layers producing at least one visible shoot. Regeneration (presence/absence) per alginate layer was analyzed using a generalized linear model (GLM) with binomial error distribution and logit link function. Shoot regenerated number per alginate layer was analyzed using a GLM with Poisson distribution and log link function. Culture medium and light quality were included as fixed factors. For the enzymatic solution assay, the data were analyzed through the one-way ANOVA with four biological replicates. For protoplast culture experiments (total response frequency, microcalli, plating efficiency, and colony-formation efficiency), a factorial design was applied and data were analyzed though the two-way ANOVA considering culture medium (Protocol 1 vs. Protocol 2) and light treatment (control, blue, and red + far-red) as factors, with five biological replicates per treatment combination (one alginate layer per Petri dish). Prior to the ANOVA, the data were tested for normality and homogeneity of variances. When significant differences were detected, means were separated using Duncan’s multiple range test at *p* ≤ 0.05. Rooting and multiplication data were analyzed using the one-way ANOVA with five biological replicates per treatment. All statistical analyses were performed using the SPSS software (IBM SPSS Statistics 25.0, IBM Corp., Armonk, NY, USA).

## 5. Conclusions

**Figure 8:**
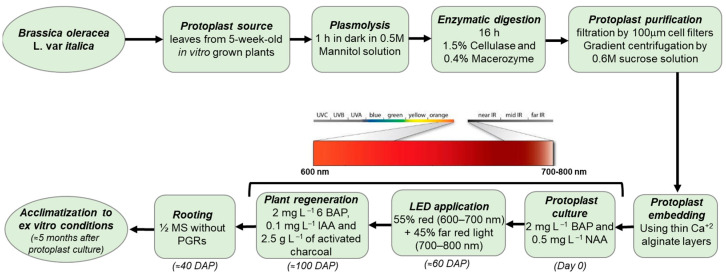
Flowchart showing the subsequent steps of the protoplast-to-plant regeneration protocol used in *Brassica oleracea* var. *italica*. Abbreviations: DAP (days after plating).

In this work, we established an efficient protoplast-to-plant regeneration system for broccoli (*Brassica oleracea* var. *italica* cv. Claremont) and identified light quality, specifically red + far-red LED illumination, as a key promotive factor in the regeneration process. A schematic summary of the optimized protoplast-to-plant regeneration protocol is provided in Figure 8. An optimized enzyme solution containing 1.5% Cellulase R-10 and 0.4% Macerozyme R-10 provided the best balance between protoplast yield and viability and was selected for all subsequent experiments. Under these conditions, alginate-embedded protoplasts regenerated most efficiently when cultured with Protocol 1 under red LED or dark conditions, whereas blue light consistently reduced microcolony formation, plating efficiency, and overall regeneration performance. De novo shoot regeneration was obtained under all light treatments except blue LED in the control medium and was maximized when red + far-red light was combined with a charcoal-containing medium, reflecting the additive contribution of spectral quality and culture composition. Shoot multiplication was not significantly affected by the BAP concentrations tested, and root formation occurred more efficiently in the hormone-free medium than in auxin-supplemented media, suggesting that a relatively simple hormonal regime is sufficient at later stages. Overall, our results demonstrate that red LED light can be used as an effective promoter of protoplast-derived regeneration in broccoli and, to our knowledge, provide one of the first reported examples of LED-assisted protoplast-to-plant regeneration in broccoli. The protocol developed here, capable of producing whole plants within approximately 5 months, offers a practical platform for CRISPR-based genome engineering, somatic hybridization, and other advanced breeding strategies in *B. oleracea*. Future work should extend this approach to additional genotypes and light spectra and investigate the underlying metabolic and molecular responses to red + far-red light that support enhanced regenerative competence.

## Figures and Tables

**Table 1:** Effect of LED light and culture media (protocol 1 or 2) on colony-formation efficiency and plating efficiency at 45 DAC in broccoli cv. Claremont protoplasts.

Light		Colony-Formation Efficiency (%)	Plating Efficiency (%)
Control	Protocol 1	12.13 ± 2.90 a	8.60 ± 1.87 a
Blue		5.75 ± 2.45 b	4.31 ± 1.42 b
Red + far-red		11.26 ± 3.80 a	8.69 ± 2.61 a
Control	Protocol 2	8.16 ± 2.10 ab	6.07 ± 1.29 ab
Blue		3.66 ± 0.56 b	2.96 ± 0.50 b
Red + far-red		10.84 ± 3.12 a	8.49 ± 2.51 a
Light		0.06 (ns)	0.03 (*)
Medium		0.34 (ns)	0.38 (ns)
Light × medium		0.81 (ns)	0.82 (ns)

All data are expressed as mean ± standard error of mean (*n* = 5). Different letters show significant differences among LED light treatments according to Duncan test (*p* ≤ 0.05). Asterisks indicate the level of statistical significance of main effects in the two-way ANOVA. Significance levels are coded as ns (non-significant), * (*p* < 0.05).

**Table 2:** Shoot number and regeneration frequency of broccoli cv. Claremont protoplast-derived colonies under different light conditions and culture media.

Factor	Level	Shoots (*n*.)	Regeneration (%)
Medium	Control	0.15 ± 0.09 b	14.0 ± 9.0 a
	Activated charcoal	0.66 ± 0.23 a	45.0 ± 15.0 a
Light	Control	0.23 ± 0.14 b	27.0 ± 14.9 ab
	Blue	0.16 ± 0.12 b	8.0 ± 8.0 b
	Red + FR	0.86 ± 0.31 a	62.0 ± 16.6 a

All data are expressed as estimated marginal means ± standard error of mean (*n* = 5). Different letters within columns indicate significant differences among factor levels according to generalized linear model pairwise comparisons (*p* ≤ 0.05).

**Table 3:** Percentage of rooting, number of roots per shoots, and root length (cm) from broccoli cv. Claremont regenerated plants.

Rooting Medium	Rooting (%)	Roots per Shoot (*n*.)	Root Length (cm)
Hormone-free medium	100.00 ± 0.00 a	10.75 ± 1.54 a	9.15 ± 1.45 a
NAA	100.00 ± 0.00 a	4.22 ± 0.44 b	1.99 ± 0.11 b
IBA	66.67 ± 19.25 a	2.78 ± 0.78 b	2.37 ± 0.59 b

All data are expressed as mean ± standard error of mean (*n* = 5). Different letters show significant differences among rooting media according to Duncan test (*p* ≤ 0.05).

## Data Availability

The raw data supporting the conclusions of this article will be made available by the authors on request.

## Supplementary Materials

The following supporting information can be downloaded at: https://www.mdpi.com/article/10.3390/plants15060905/s1, Table S1. Composition of culture media used for protoplast proliferation, callus development, shoot regeneration and rooting in broccoli (*Brassica oleracea* var. *italica* cv. Claremont).
