# Supplementary material for: Red and Far-Red LED Lighting Enhances Protoplast-to-Plant Regeneration in Broccoli (Brassica oleracea var. italica)
Source: Plants (Basel). 2026 Mar 14;15(6):905. doi: 10.3390/plants15060905 (PMC13030773; doi:10.3390/plants15060905)
Supplement: Supplementary file 1 [file plants-15-00905-s001.zip › plants-4193695-supplementary.pdf]

Table S1. Composition of culture media used for protoplast proliferation, callus development, shoot regeneration and rooting in broccoli (*Brassica oleracea* var. *italica* cv. Claremont).

**Protoplast proliferation medium 1 (PPM-1)**	**Concentration**	**Reference**
Gamborg B5 with vitamins	Basal medium	[1]
Sucrose	20 g L^{-1}	
Myo-inositol	60 g L^{-1}	
6-BAP	2 mg L^{-1}	
NAA	0.5 mg L^{-1}	
**Protoplast proliferation medium 2 (PPM-2)**	**Concentration**	**Reference**
1/2 Gamborg B5 with vitamins	Basal medium	[2]
Glucose	20 g L^{-1}	
D-Manitol	70 g L^{-1}	
BAP	1 mg L^{-1}	
NAA	1 mg L^{-1}	
2,4-D	0.25 mgL^{-1}	
**Callus proliferation medium 1 (CPM-1)**	**Concentration**	**Reference**
Gamborg B5 with vitamins	Basal medium	[1]
Sucrose	20 g L^{-1}	
6-BAP	2 mg L^{-1}	
NAA	0.5 mg L^{-1}	
**Callus proliferation medium 1 (CPM-2)**	**Concentration**	**Reference**
1/2 Gamborg B5 with vitamins	Basal medium	[2]
Glucose	20 g L^{-1}	
D-Manitol	40 g L^{-1}	
BAP	1 mg L^{-1}	
NAA	0.2 mg L^{-1}	
**Shoot regeneration media (SRM-1)**	**Concentration**	**Reference**
2/3 MS macro- microelements with vitamins	Basal medium	This study
Sucrose (3%)	30 g L^{-1}	
BAP	2 mg L^{-1}	
IAA	0.1mg L^{-1}	
Agar	7 g L^{-1}	
**Shoot regeneration media (SRM-2)**	**Concentration**	**Reference**
2/3 MS macro- microelements with vitamins	Basal medium	This study
Sucrose (3%)	30 g L^{-1}	
BAP	2 mg L^{-1}	
IAA	0.1mg L^{-1}	
Agar	7 g L^{-1}	
Activated charcoal	2.5 g L^{-1}	
**Rooting media (RM-1)**	**Concentration**	**Reference**
1/2 MS macro- microelements with vitamins	Basal medium	This study
Sucrose	10 g L^{-1}	
Agar	7 g L^{-1}	
**Rooting Media (RM-2)**	**Concentration**	**Reference**
1/2 MS macro- microelements with vitamins	Basal medium	This study
Sucrose	10 g L^{-1}	
NAA	1 mg L^{-1}	
Agar	7 g L^{-1}	
**Rooting media (RM-3)**	**Concentration**	**Reference**
1/2 MS macro- microelements with vitamins	Basal medium	This study
Sucrose	10 g L^{-1}	
NAA	1 mg L^{-1}	
Agar	7 g L^{-1}	

References:

1. Jeong, Y.Y.; Lee, H.-Y.; Kim, S.W.; Noh, Y.-S.; Seo, P.J. Optimization of Protoplast Regeneration in the Model Plant Arabidopsis Thaliana. *Plant Methods* **2021**, 17, 21.

2. Hussain, M.; Li, H.; Badri Anarjan, M.; Lee, S. Development of a General Protoplast-Mediated Regeneration Protocol for Brassica: Cabbage and Cauliflower as Examples. *Hortic. Environ. Biotechnol.* **2023**, doi:10.1007/s13580-023-00557-4.
